# Tumor progression locus 2 ablation suppressed hepatocellular carcinoma development by inhibiting hepatic inflammation and steatosis in mice

**DOI:** 10.1186/s13046-015-0254-2

**Published:** 2015-11-11

**Authors:** Xinli Li, Chun Liu, Blanche C. Ip, Kang-Quan Hu, Donald E. Smith, Andrew S. Greenberg, Xiang-Dong Wang

**Affiliations:** Nutrition and Cancer Biology Laboratory, Jean Mayer USDA Human Nutrition Research Center on Aging at Tufts University, 711 Washington Street, Boston, MA 02111 USA; Comparative Biology Unit, Boston, MA USA; Obesity and Metabolism Laboratory, Jean Mayer USDA Human Nutrition Research Center on Aging at Tufts University, Boston, MA 02111 USA; School of Public Health, Medical College of Soochow University, Suzhou, Jiangsu 215123 PR China

**Keywords:** TPL2, HCC, Tumorigenesis, Inflammation, Steatosis

## Abstract

**Background:**

Tumor progression locus 2 (TPL2), a serine-threonine kinase, functions as a critical regulator of inflammatory pathways and mediates oncogenic events. The potential role of *Tpl2* in nonalcoholic fatty liver disease (NAFLD) associated hepatocellular carcinoma (HCC) development remains unknown.

**Methods:**

Both wild-type and *Tpl2* knockout male mice were initiated by a hepatic carcinogen (diethylnitrosamine, i.p. with a single dose of 25 mg.kg^−1^)at 2 weeks of age, and then were given the high carbohydrate diet feeding to induce hepatic steatosis, inflammation, adenoma and HCC for 24 weeks.

**Results:**

*Tpl2* knockout mice had significantly lower incidences of liver tumor and developed hepatocellular adenoma only, which is contrast to wild-type mice where they all developed HCC. *Tpl2* knockout mice had significantly down-regulated phosphorylation of JNK and ERK, and levels of mRNA expression of pro-inflammatory cytokines (*Il-1β, Il-18, Mcp-1* and *Nalp3*), which correlated with the reduced incidence and number of hepatic inflammatory foci. Furthermore, *Tpl2* ablation resulted in decreased hepatic steatosis and expression of de novo lipogenesis related markers (ACC, SCD1, SREBP1C and AKT phosphorylation), as well as reduction of endoplasmic reticulum stress biomarkers PERK and eIF-2a.

**Conclusion:**

The study revealed for the first time that *Tpl2* plays a significant role in promoting HCC development by its pro-inflammatory effect, which suggested that *Tpl2* could be a molecular target for HCC prevention.

## Background

Hepatocellular carcinoma (HCC) is the sixth most prevalent human malignancy in the world and the third leading cause of cancer-related mortality [1]. Relative 5-year survival rate of HCC is only 15 %, which emphasizes the importance of primary prevention of HCC. Chronic infection by hepatitis B and hepatitis C virus, exposure to aflatoxin, alcoholic injury and genetic disorders have proven to play a critical role in the development of HCC [2], however, the etiology remains unknown in almost 50 % of HCC patients. Recent studies suggest that nonalcoholic fatty liver disease (NAFLD) is associated with an increased risk of HCC [3, 4], but it remains unclear whether NAFLD is a causative factor for HCC [5].

Steatosis is the initial stage of NAFLD, which can progress into more pathological stages including nonalcoholic steatohepatitis (NASH), fibrosis and cirrhosis, with the result of the increased risk for HCC development. Previous studies had indicated the significant contribution of NASH to HCC development, where pro-inflammatory cytokine and chemokines favors malignant transformation of hepatocytes by providing a tumor microenvironment [6, 7]. Inflammatory cascades through interactions of numerous signaling pathways progressively stimulated hepatocyte proliferation and apoptosis [8]. However, the potential causal relationships among inflammation, steatosis and HCC development need more supporting evidence.

Tumor progression locus 2 (TPL2), a serine-threonine kinase, functions as a critical regulator of inflammatory pathways and mediates oncogenic events by phosphorylating its downstream targets extracellular signal regulated kinases (ERKs), c-Jun N-terminal kinases (JNKs) and P38 [9] and subsequently up-regulating the production of tumor necrosis factor-α (TNF-α) and interleukin-1β (IL-1β) [10]. The role of *Tpl2* in acute and chronic inflammatory disorders and the determination of cellular death/survival ratios in the inflammatory microenvironment had been well-documented [11]. Our previous work demonstrated that whole-body ablation of *Tpl2* attenuates high fat diet (HFD) induced hepatic inflammatory lesions compared to wild-type control mice, with a concomitant significant reduction in hepatic inflammatory genes expression [12]. However, the potential role of *Tpl2* in tumorigenesis remains inconsistent [11, 13]. Several reports supported its oncogenic role in breast cancer [14], lymphoma [15] and prostate cancer [16], while others had suggested an anti-oncogenic activity in lung [17], colitis-associated tumorigenesis [18] and skin tumorigenesis [19]. This inconsistency could be attributed to the complexity of *Tpl2*’s role in terms of specific organs, different stages of carcinogenesis or the animal models used. However, there had no reports investigating the potential contribution of *Tpl2* to HCC development by using *Tpl2* knockout mouse model to date.

In the present study, we investigate the role of *Tpl2* and its potential mechanisms in the development of hepatic steatosis, inflammation and tumors including HCC. Both wild-type and *Tpl2* knockout male mice were initiated with [diethylnitrosamine (DEN)] at 2 weeks of age, and 4 weeks later, both groups mice were given the high carbohydrate diet (HCD) feeding for 24 weeks to induce hepatic steatosis, inflammation and HCC.

## Methods

### Animals, diet, carcinogen and study design

*Tpl2* knockout mice were provided by Dr. Philip Tschilis (Tufts University) and backcrossed into C57BL/6J mice for >10 generations, as previously described [12, 20]. Genotype of animals was verified at weaning and again before the animals were killed at the conclusion of the experiment. *Tpl2* mRNA expression was detected in wild-type but not in *Tpl2* knockout mice (data not shown). All male C57BL/6J wild-type mice and *Tpl2* knockout mice were injected i.p. with a single dose of 25 mg.kg^−1^ BW filter-sterilized, > 99.9 % purity DEN (Sigma-Aldrich, St. Louis, MO) at 2 weeks after birth, as previously described [21]. At 6 weeks of age, both wild-type (n = 26) and *Tpl2* knockout (n = 20) mice were housed individually and fed a powdered, high carbohydrate, low fat diet [HCD, 12 % fat, 22 % protein, 66 % carbohydrates based on total caloric content (Bio-Serv, Flemington, NJ)] ad libitum for 24 weeks, as previously described [22]. Mice were killed by terminal exsanguination under deep isoflurane anesthesia followed by vital organ removal. All animal protocols and procedures were conducted under the approval of the Institutional Animal Care and Use Committee at the Jean Mayer USDA Human Nutrition Research Center on Aging at Tufts University. All animals received human care and that study protocols comply with the institution's guidelines.

### Histopathology procedures and evaluation

Briefly, two investigators, blinded to the treatment groups, identified/counted the liver tumors (tumor incidence and multiplicity) on the surface of liver. The left lobe of the mouse liver was fixed in 10 % buffered formalin solution (Thermo Fisher Scientific, Waltham MA), processed and embedded in paraffin for serial sectioning for pathological analysis. The remaining lobes of the liver were divided into smaller portions, snap-frozen in liquid nitrogen and stored at -80 °C.

Five-micrometer sections of liver tissue were stained with hematoxylin (H) and eosin (E) (H&E) for histopathologic examination. Two independent investigators, blinded to treatment groups, examined the sections under light microscopy and identified hepatic lesions (including hyperplasia, hepatocellular adenoma and HCC). Liver histopathology of non-tumor areas was graded for steatosis magnitude (both macro- and micro- vesicular) and liver inflammation severity (inflammatory foci) as previously described [21]. Briefly, the degree of steatosis was graded 0–3 [grading 0: <5 % (normal), 1: 5 %-25 %, 2: 26 %-50 %, 3: >51 %] based on the average percent of fat-accumulated hepatocytes per field at 100 × magnification in 20 random fields. Inflammatory foci were evaluated by the number of inflammatory cell clusters (mononuclear inflammatory cells) in 20 random fields at 100 × magnification. The twenty fields of view at 100 × magnification represented 0.63 cm^2^ and inflammatory foci counts were represented as the number of foci per cm^2^. A ZEISS microscope with a PixeLINK USB 2.0 (PL-B623CU) digital Camera and PixeLINK μScope Microscopy Software (Ottawa ON, Canada) was used for image capture for all histological analyses.

### RNA extraction and real time-PCR

RNA of liver tissue was isolated using TriPure Isolation Reagent (Roche, NJ) according to the manufacturer’s instructions. cDNA was synthesized using M-MLV reverse transcriptase (Invitrogen, Grand Island, NY). Real-time PCR reactions were carried out using SYBR green (Fast Start Universal SYBR Green Master, Roche). Relative changes in gene expression were determined using the 2^-ΔΔCt^ method and normalized to the control of actin.

### Western blot analysis

Hepatic whole cell lysate protein was extracted utilizing previously described methods [23]. Protein concentrations of the sample were assessed using the Bradford assay (Bio-Rad, Hercules CA). The protein expression bands were quantified using a densitometer (GS-710 calibrated imaging densitometer, Bio-Rad). The antibodies against acetyl-CoA carboxylase (ACC), stearoyl-CoA desaturase 1 (SCD-1), total- and phosphor- AKT (p-AKT, t-AKT), glucose regulated protein 78 (GRP78/Bip), total- and phosphor- (Thr980) PERK (t-PERK, p-PERK), total- and phosphor- (Ser51) eukaryotic translation initiation factor 2a (t-eIF2a, p-eIF2a), total- and phosphor- (Thr183/Tyr185) JNK (p-JNK, t-JNK), total- and phosphor- ERK (p-ERK, t-ERK) were all purchased from Cell Signaling (Danvers, MA). The antibodies against sterol regulatory element-binding protein-1c (SREBP-1c) and C/EBP homologues protein (CHOP) were purchased from Santa Cruz Biotechnology (Dallas, TX).

### Statistical analyses

Data are presented as mean ± standard deviation (SD) for animal body weights, liver tumor numbers per animal and inflammatory foci, and nonparametric test was performed except for the comparison of the final body weights which was conducted using an independent t-test. The incidence of hepatic lesions and inflammation foci was compared by Chi-squares test. Steatosis grading was presented as median (grading range) and a non-parametric test was performed. Data are presented as mean ± standard error of the mean (SEM) for mRNA and protein levels, and t-test was conducted. SPSS software was used for all statistical analysis, and *P* < 0.05 indicated the significant difference when comparing between wild-type and *Tpl2* knockout mice.

## Results

### Effect of *Tpl2* ablation on body weight, liver weight and hepatic tumorigenesis

**Fig. 1:**
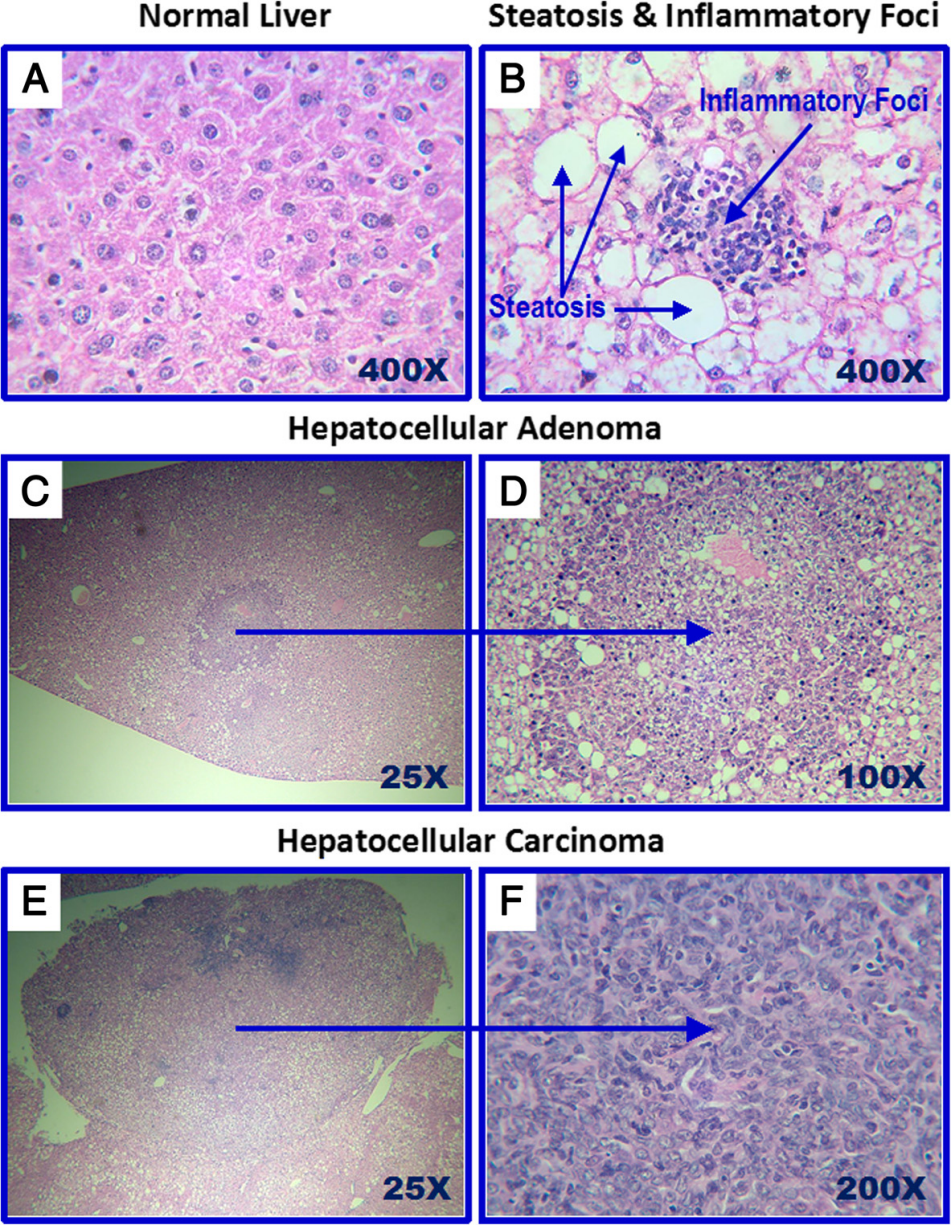
Representative pathologic lesions in livers. Hepatic lesions were assessed by H&E staining. Upper panel: Normal (Left); Steatosis and inflammatory foci (Right); Middle Panel: Hepatocellular adenoma (low magnification at x 25 and x100); Lower panel: HCC (low magnification at x25 and x200)

*Tpl2* knockout mice had significantly lower liver weight and final body weight than those of wild-type control, although there had no significant difference in the ratio of liver weight/body weight between two groups (Table 1). The tumor incidence on the surface of liver in *Tpl2* knockout mice was significantly lower than wild-type mice (75 % vs 100 % respectively, *P* < 0.05, Table 1). The number of hepatic tumor had no statistical difference between those two groups. The pathological analysis demonstrated that all wild-type mice (26 out of total 26 mice) developed hyperplasia, hepatocellular adenoma (Fig. 1c, 1d) and HCC (Fig. 1e, 1f) after the 24-week HCD feeding (Table 1). In contrast, the *Tpl2* knockout mice with positive tumor on the surface of the liver (15 out of 20) developed only hyperplasia and hepatocellular adenoma, and no HCC detected (Table 1).

**Table 1:** Study outcomes

Index	Wild type group		*Tpl2* knockout group	
Animal number (n)	26		20	
Final body weight (g)	41.2 ± 3.2		38.1 ± 5.9*	
Liver weight (g)	2.5 ± 0.8		2.1 ± 0.3*	
Liver/body weights (%)	6.2 ± 2.4		5.6 ± 1.1	
Incidence of liver tumor (%)	100 % (26/26)		75 % (15/20)*	
Liver tumor number/per animal	11 ± 6		10 ± 5	
Histopathology of hepatic lesion^#^	n	%	n	%
Hyperplasia	26	100	3	15*
Hepatocellular adenoma	26	100	12	60*
Hepatocellular carcinoma	26	100	0	0*
Incidence of inflammation foci (%)	65.4% (17/26)		20 % (4/20)*	
Inflammation foci number (cm^2^)	2.2 ± 2.4		0.6 ± 1.4*	
Hepatic steatosis (median)	2		1*	
Hepatic steatosis grading	n	%	n	%
0	1	3.8	8	40
1	2	7.7	4	20
2	15	57.7	4	20
3	8	30.8	4	20

^#^Each animal has more than one type of lesions

Values are expressed as means ± SD. An Independent t-test was performed except the comparison of incidence of liver tumor and inflammatory foci between WT and Tpl2 KO mice, which is conducted by nonparametric test. For steatosis, 20 images at 100 × magnification were captured for each section and blindly evaluated twice to determine grade of steatosis (both macro- and micro-vesicular) by two blinded investigators. The degree of steatosis was graded 0-3 based on the area of the liver section occupied by fat vacuoles. Data are presented as median (grading range) and Nonparametric test was used to test for statistical significance between groups for ordinal variable (liver steatosis score)

For each given row, * indicates a significant difference between groups (P < 0.05)

### *Tpl2* ablation decreased hepatic inflammatory responses and suppressed the activation of JNK and ERK signaling molecules

**Fig. 2:**
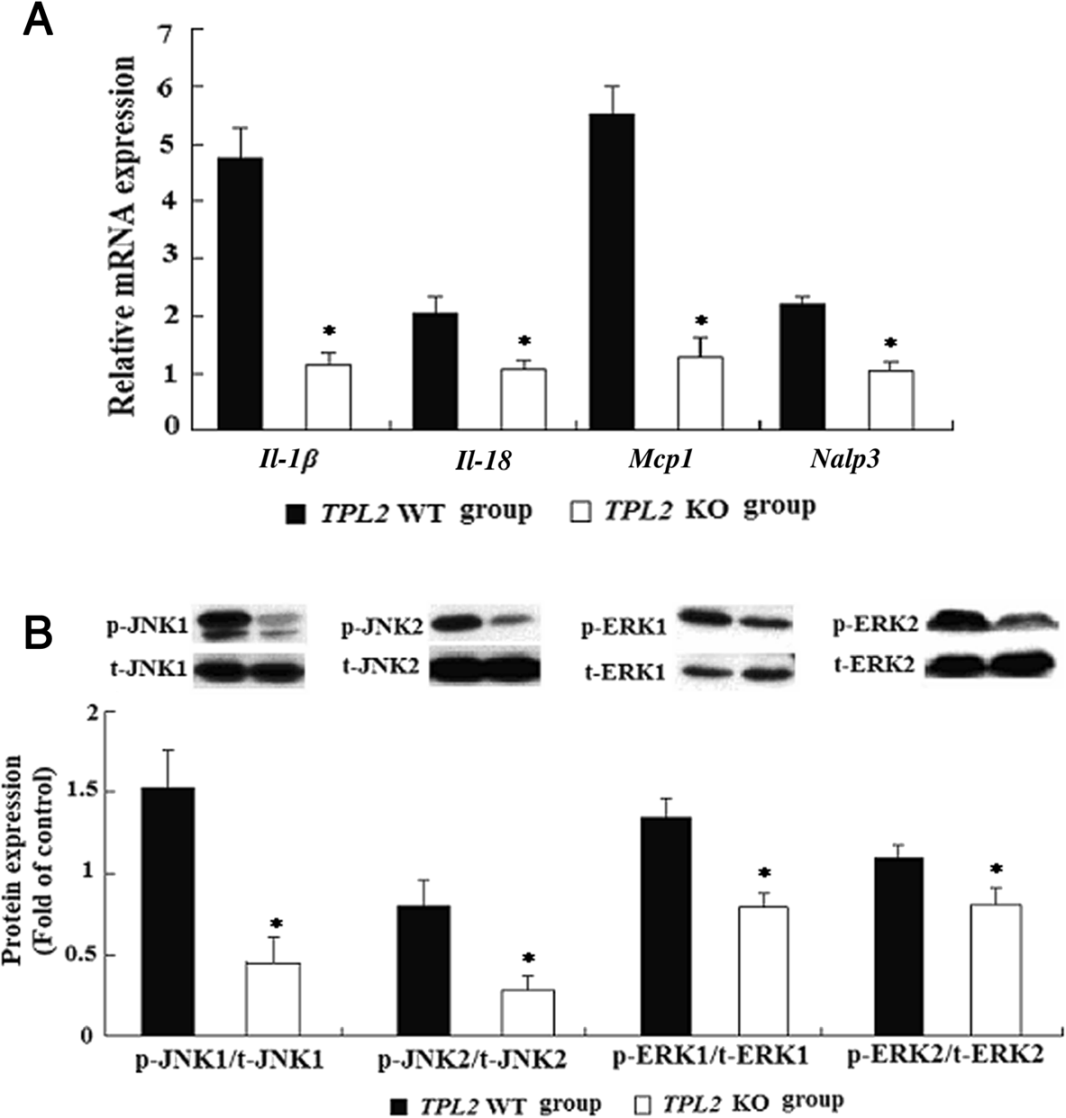
Effect of *Tpl2* ablation on hepatic mRNA expression of genes related to inflammation (**a**) and protein phosphorylations of JNK1/2 and ERK1/2 (**b**). **a** mRNA expression of genes related to inflammatory and macrophage markers in liver tissue in mice were detected by RT-PCR analysis. Values are expressed as mean ± standard error of the mean (SEM). Actin was used as the control. **b** Proteins expression of JNK1/2 and ERK1/2 from liver tissue of *Tpl2* knockout or wild-type mice were detected by western blotting analysis. Values are mean ± standard error of the mean (SEM). *Comparing with *Tpl2* wild type group. Insets: Representative pictures of western blotting analysis

Hepatic inflammatory foci was detected in 65 % of the wild-type mice but only in 20 % of the *Tpl2* knockout mice (*P* < 0.05; Table 1, Fig. 1b). *Tpl2* knockout mice had significantly less hepatic inflammatory foci in contrast to wild-type mice (0.6 *vs* 2.2, *P* < 0.05; Table 1), accompanying with lower mRNA level of genes [*Il-1β*, interleukin-18 (*Il-18*), monocyte chemotactic protein 1 (*Mcp1*), NACHT, LRR and PYD domains-containing protein 3 (*Nalp3*) related to hepatic inflammation (Fig. 2a)]. In addition, there was a significant decrease in the phosphorylation of JNK1/2 and ERK1/2, the downstream targets of TPL2-mediated inflammation signaling, in *Tpl2* knockout mice as compared with wild-type mice (Fig. 2b).

### *Tpl2* ablation alleviated hepatic steatosis, and down-regulated protein and mRNA expression of molecules involved in de novo lipogenesis (DNL) and endoplasmic reticulum (ER) stress

**Fig. 3:**
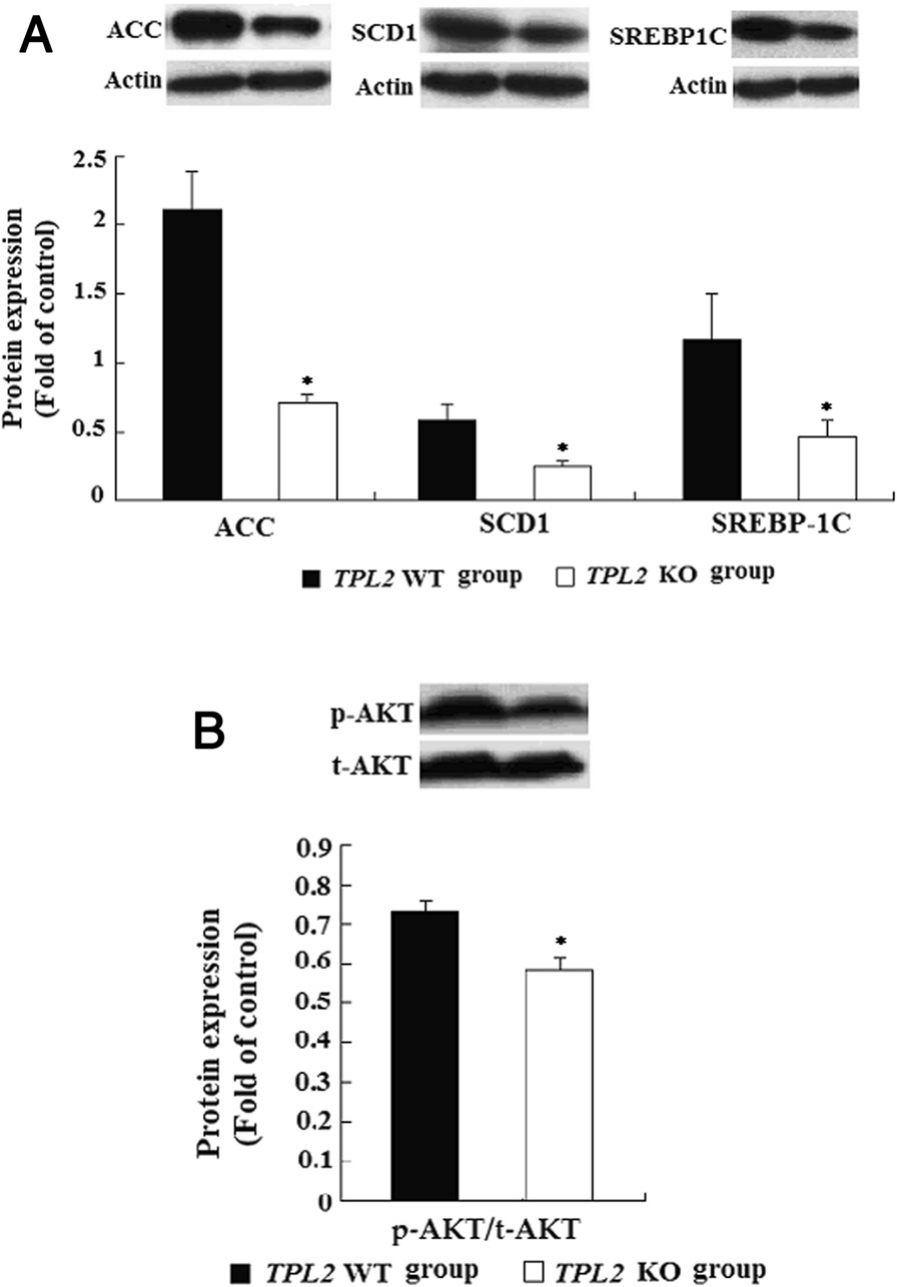
Effect of *Tpl2* ablation on hepatic protein expressions related to lipid metabolism (**a**) and AKT phosphorylation (**b**). Proteins expression related to lipogenesis (**a**) and AKT phosphorylation (**b**) from liver tissue were determined utilizing Western blotting analysis. Data are presented as mean ± standard error of the mean (SEM). Actin was used as the control. *Comparing with *Tpl2* wild type group. Insets: Representative pictures of western blotting analysis

*Tpl2* knockout mice had lower steatosis grades compared to wild-type mice (Table 1). Only one of 26 wild-type mice (3.8 %) had a steatosis grading of 0 compared to 8 out of 20 *Tpl2* knockout mice (40 %). In contrast, 15 out of 26 wild-type mice (57.7 %) developed steatosis grade of 2 as compared to 4 out of 20 *Tpl2* knockout mice (20 %). Steatosis grades were statistically different between the 2 groups (medians of 2 *vs*. 1 for wild-type *vs. Tpl2* knockout, respectively, *P* < 0.05, Table 1). In contrast to wild-type mice, *Tpl2* knockout mice had significantly decreased proteins expression of ACC and SCD1, two lipogenic proteins (Fig. 3a), and decreased protein expression of SREBP1C (Fig. 3a), which is one of the transcription factors that regulates expression of genes involved in DNL. Meanwhile, we had detected the decreased expression of phosphorylated AKT (Fig. 3b), which is consistent with the decreased SREBP1c expression (Fig. 3a) and hepatic steatosis (Table 1) in *Tpl2* knockout mice.

**Fig. 4:**
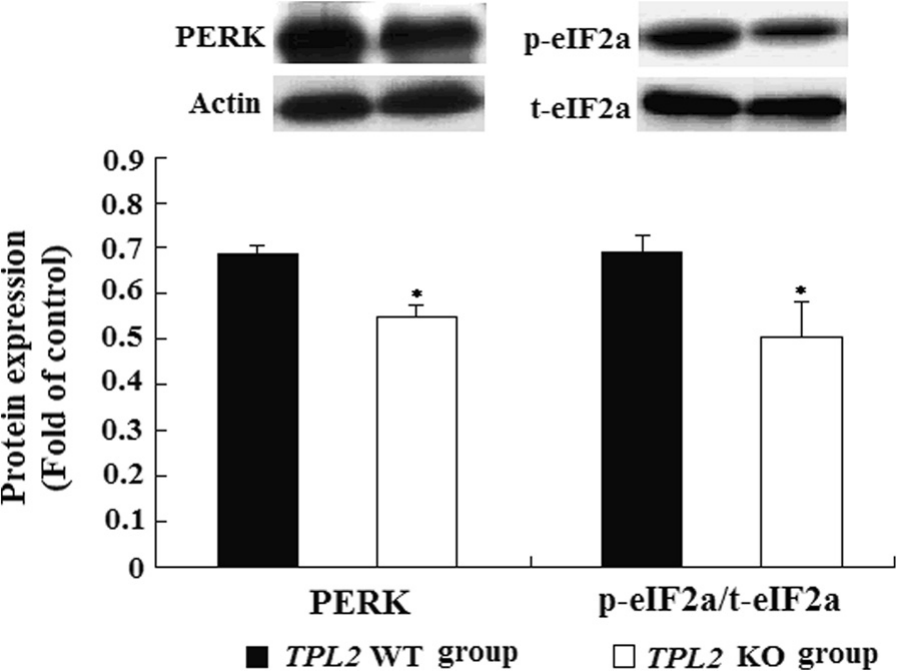
Effect of Tpl2 ablation on hepatic ER stress biomarkers. Proteins expression related to ER stress were examined by western blotting analysis. Values are mean ± standard error of the mean (SEM). Actin was used as the control. *Comparing with *Tpl2* wild type group. Insets: Representative pictures of western blotting analysis

Increased ER stress can promote HCC development and progression by activating the fibrogenic activity of hepatic stellate cells with subsequent liver cirrhosis [24]. In the present study, *Tpl2* knockout mice had decreased hepatic PERK and eIF2α phosphorylation compared to wild-type mice (Fig. 4), but *Tpl2* ablation did not alter the expression of the chaperon factor GRP78 that attenuates ER stress or PERK mediated pro-apoptotic protein CHOP (data not shown).

## Discussion

The role of hepatic inflammation induced by dietary factors, such as HCD and HFD, in promoting DEN-initiated HCC development had been demonstrated in previous studies [6, 22, 25]. The present study, for the first time, revealed the significantly lower incidence of hepatic tumor with no HCC development in HCD-fed, *Tpl2* knockout mice in contrast to wild-type mice which all developed HCC. The significant difference in tumor pathological types between *Tpl2* knockout mice and wild-type mice supported the critical role of *Tpl2* as a promoter in tumor progression from hepatic hepatocellular adenoma to HCC. Furthermore, we provided a strong evidence that the TPL2 ablation decreased hepatic inflammatory response and hepatic steatosis in *Tpl2* knockout mice. These effects could inhibit the malignant transformation of hepatocytes and the progression of liver tumor by suppressing tumor-promoting microenvironment and alleviating the malignant effects of dys-regulation of lipid metabolism on HCC.

TPL2 mediated inflammatory response by phosphorylating ERK and JNK, two downstream targets of TPL2 signaling pathway. In our study, the significant lower protein levels of hepatic p-JNK and p-ERK in *Tpl2* knock out mice was consistent with the decreased hepatic inflammation, which supported the role of activated ERK (p-ERK) and JNK (p-JNK) in mediating the pro-inflammatory effect of *Tpl2*. The decreased hepatic inflammation induced by HCD feeding supported by the fewer hepatic inflammatory foci detected and the lower levels of inflammatory cytokine expression of *Il-1β*, *Il-18*, *Mcp-1* and *Nalp3* in *Tpl2* knockout mice, as compared with wide type mice. This is also in agreement with our previous study that *Tpl2* knockout mice fed HFD had lower levels of inflammation compared to wild-type mice [12]. The present work further indicated the role of *Tpl2* in mediating hepatic inflammation and HCC development induced by HCD.

Elevated hepatic DNL could promote hepatic steatosis [26]. Interestingly, in our present study, *Tpl2* ablation resulted in a significant decrease of hepatic steatosis, and the down-regulated protein expression of genes related to DNL, such as ACC and SCD1, which was associated the decreased protein expression of AKT phosphorylation and SREBP-1C.

Previous studies have suggested that AKT activation is essential and sufficient to stimulate DNL and lipid accumulation through the induction of SREBP-1C [27, 28]. Furthermore, the promoting role of dysregulated lipid metabolism [29] and lipogenesis induced by activated AKT in HCC development had been documented [30]. Thus, the decrease in steatosis and AKT activation could further explain the decreased HCC in *Tpl2* knockout mice.

However, the exact role of *Tpl2* in regulating the genes related to DNL is unclear. It has been reported that the regulation of lipid metabolism and hepatic steatosis mediated by the activation of JNK, the downstream target of TPL2, is associated with activated ER stress, especially in the PERK-eIF2a pathway [31]. Our observation of the down-regulated expression of p-JNK, PERK and p-eIF2a in *Tpl2* knockout mice with the decreased hepatic steatosis supported the involvement of ER stress in TPL2/JNK mediated steatosis. Recent reports have demonstrated that ER stress is closely associated with hepatic lipogenesis with elevated DNL [26, 32, 33], and population-based studies also support the positive regulation of ER stress in hepatic lipogenesis [34, 35]. Since newly synthesized unfolded proteins in the ER is a major cause of activated ER stress and activated ER stress could further induce lipogenesis, the vicious cycle could result in the progression of steatosis [21]. In our present study, the deceased expression of the lipogenic enzymes ACC, SCD1 and SREBP-1C could decrease the impact of protein folding in the ER, and alleviate ER stress in *Tpl2* knockout mice. PERK-mediated signaling can also promote apoptosis through inducing pro-apoptotic CHOP expression, the no significant difference of the protein level of CHOP suggested that HCD induced expression of PERK predominantly mediated lipogenesis, but not pro-apoptotic effects in our present study. Furthermore, the expression of GRP78, a chaperone protein that attenuates ER stress [36], were not different between the *Tpl2* knockout and wild-type mice, combining with our observation that *Tpl2* knockout mice had relatively lower levels of PERK and p-eIF2a, the downstream molecular of GRP78, than wild-type mice, we therefore concluded that *Tpl2* mediated hepatic lipogenesis by targeting the axis of TPL2/JNK/ER stress/p-eIF2a, the downstream of GRP78.

Our previous study had shown that HCD feeding promoted DEN-initiated HCC development accompanying with the induction of the hepatic ER stress-mediated PERK activation, which subsequently induced the elevated expression of pro-survival markers AKT and ERK1/2 [22]. Our present study demonstrated that *Tpl2* ablation decreased ER stress mediated PERK expression and eIF-2a activation, which might account for the decreased tumor incidence. Since ER-dependent cell fate is associated with the activation of JNK/ERK [37], and the pro-survival role of activated ERK can be mediated by AKT phosphorylation and the involvement of PERK/eIF2a signaling [38–40], thus, in the present study, the down-regulated activation of ERK, AKT and PERK/eIF2a provided further explanation for the decreased incidence of HCC in *Tpl2* knockout mice. The exact relationship between TPL2 and ER stress requires further investigation.

In summary, our present study demonstrated that *Tpl2* played significant role in DEN-initiated, NAFLD associated HCC development by using *Tpl2* knockout mouse model. Both TPL2/ERK/JNK axis mediated hepatic inflammation and TPL2/JNK/ERS/p-eIF2a axis mediated hepatic lipogenesis synergistically promoted HCC development. These data provided strong molecular evidence supporting *Tpl2* as a promoter in HCC development. Interestingly, it has been shown that luteolin, one of the common phytonutrients present in celery, parsley, broccoli and herbal spices, could target *Tpl2* and inhibit its activity in vitro [41]. Recent in vivo studies also supported the preventive effects of luteolin on DEN-initiated alcohol-promoted hepatic carcinogenesis in mice [42], and DEN-initiated HCC development in rats [43]. Taking all into consideration, dietary or pharmacologic interventions targeting *Tpl2* could be a potential direction for HCC prevention in the future.

## Conclusions

This is the first study to report that tumor progression locus 2 (Tpl2) plays a significant role in promoting nonalcoholic fatty liver disease and hepatocellular carcinoma (HCC) development. Targeting Tpl2 might be a potential direction for HCC prevention.

## Abbreviations

HCC: hepatocellular carcinoma
NAFLD: non-alcoholic fatty liver disease
TG: triglycerides
NASH: nonalcoholic steatohepatitis
TPL2: tumor progression locus 2
MAP3K8: MAP3 kinase 8
ERKs: extracellular signal regulated kinases
JNKs: c-jun N-terminal kinases
TNF-α: tumor necrosis factor-α
IL-1β: interleukin-1β
HFD: high fat diet
DEN: diethylnitrosamine
HCD: high carbohydrates diet
AKT: protein kinase B
ER: endoplasmicreticulum
PERK: PKR-like kinase
ACC: acetyl-CoA carboxylase
SCD: stearoyl-CoA desaturase
p-: phosphorylated
t-: total
GRP78: Glucose regulated protein 78
eIF2a: eukaryotic translation initiation factor 2a
SREBP-1c: sterol regulatory element-binding protein-1c
CHOP: C/EBP homologues protein
SD: standard deviation
SEM: standard error of the mean
IL-18: Interleukin-18
MCP-1: monocyte chemotactic protein 1
NALP3: NACHT, LRR and PYD domains-containing protein 3
DNL: de novo lipogenesis
